# Pixel-Level and DNA-Level Image Encryption Method Based on Five-Dimensional Hyperchaotic System

**DOI:** 10.3390/e27121221

**Published:** 2025-12-01

**Authors:** Min Zhou, Xin Li, Wenqi Du, Jianming Li, Zhe Wei

**Affiliations:** School of Computer Science and Artificial Intelligence, Civil Aviation Flight University of China, Guanghan 618307, China; lizon@cafuc.edu.cn (X.L.);

**Keywords:** image encryption, chaotic system, DNA sequence operations, joint diffusion, intra-block diffusion, security analysis

## Abstract

Images, as carriers of rich information, are generated, stored, and transmitted in various forms across diverse scenarios. It has become an important issue in the field of information security today to encrypt images to ensure information security. To address this issue, this paper proposes a Pixel-Level and DNA-Level Image Encryption Method Based on a Five-Dimensional Hyperchaotic System, named PD5H. The proposed method combines a five-dimensional chaotic system, a novel pixel-block internal diffusion method, and a new flow diffusion method integrating Pixel-Level and DNA-Level encryption, hereinafter referred to as ‘joint diffusion’. The improved 5D chaotic system can generate highly complex and unpredictable chaotic sequences. The intra-block diffusion process utilizes the internal information of the image to perform preliminary diffusion and reduce pixel correlation. The joint diffusion process can effectively employ various encryption methods to encrypt images with different step sizes at the bit level. PD5H has a large key space, extremely low image correlation, a uniform ciphertext pixel distribution, an excellent ciphertext entropy value (>7.999), and strong resistance to differential attacks. It also demonstrates strong resistance to data loss. The security analysis confirms that PD5H demonstrates excellent performance in color image encryption and can effectively resist various common attacks.

## 1. Introduction

With the widespread application of generative artificial intelligence, a large number of images are generated, transmitted, stored, and used in various aspects of society. For the sake of information security, image encryption methods are widely used in the storage and transmission of images. However, due to the unique characteristics of images, such as high redundancy and strong pixel correlation, traditional encryption methods are not suitable for image encryption. Chaotic systems are widely used in image encryption due to their many advantages, such as pseudo-randomness, ergodicity, and extreme sensitivity to control parameters and initial values [1,2,3,4]. 

Many studies have established various image encryption methods based on chaotic systems and combined them with various other methods, for example, encryption algorithms that combine chaotic systems and neural networks [5,6,7,8,9,10], encryption methods that combine quantum computing and chaotic systems [11,12], and methods that combine chaotic systems and compressive sensing [13,14,15].

Among them, DNA manipulation has been widely applied. DNA manipulation, due to its unique properties, encrypts images at a new level, breaking the strong correlation between adjacent pixels, and is widely used in the process of image diffusion [16,17,18,19]. The eight-base DNA manipulation is an excellent improvement on DNA manipulation in recent years, adding four artificial bases and expanding the diversity and randomness of DNA manipulation [20,21].

Image diffusion and permutation are important methods for image encryption. Permutation is the operation of changing the position of pixels through certain rules, while diffusion is the operation of using the information of chaotic sequences or its own information to change the values of pixels. Zhongyun Hua et al. proposed a method of diffusion in different color planes, expanding the range of diffusion [22]. DNA manipulation is an important diffusion method that recombines image pixels according to certain DNA rules to obtain base sequences and perform different operations on the base sequence, which generally include “ addition “, “ XOR “, “ transcription “, etc. When restored to pixels, encrypted images exhibit higher information entropy. In recent years, there have also been methods that combine diffusion and permutation operations [23,24,25,26], reducing encryption steps and improving encryption efficiency.

In addition, the idea of dividing subproblems is widely used in image encryption. There are two branches of image encryption technology based on chaos: image encryption based on stream cipher, and image encoding based on block cipher [27,28]. Splitting images by channel is the most fundamental and intuitive method [29,30,31]. Hua, Z. et al. proposed a method for processing images using Latin orthogonalization [32]. Zhang, D. et al. proposed a method of splitting images into Latin cubes, which increases the dimension of encryption and improves the randomness of encryption [33]. W. Feng et al. analyzed existing methods and improved the encryption method based on Feistel Network [34]. Y. Wang et al. proposed dividing the image into n uniform equal parts, encrypting them separately, and finally recombining them into encrypted images, which can fully utilize the multi-threaded advantages of advanced devices and greatly shorten the execution time of the algorithm [35].

In 1994, Adleman et al. first used DNA molecules to solve the Hamiltonian pathway problem, verifying the feasibility of using biomolecules as computational media and laying the foundation for subsequent DNA information processing technologies. DNA manipulation refers to the process of recombining an image into a sequence of nucleotides in a certain way, and then encrypting the base sequence by controlling the sequence, ultimately achieving better diffusion effects. In the 2010s, DNA manipulation and chaotic systems were organically combined in image encryption, giving rise to a large number of excellent encryption methods. However, at this time, base manipulation was still rare and simple, mainly relying on the randomness of chaotic systems to achieve good results. In recent years, scholars have proposed many new innovations based on this foundation. U.K. Gera et al. introduced algebraic operations into base operations [36], increasing the diversity of base operations. Yu, Jinwei et al. introduced the triploid mutation method into DNA manipulation and achieved good results, significantly reducing correlation [37]. Fan et al. introduced eight-base DNA manipulation into image encryption, revitalizing DNA base manipulation and increasing the breadth and depth of DNA manipulation [19].

These methods have their unique advantages, but they all have a common drawback of limited encryption dimensions: most methods only choose one encoding and decoding method for encryption, and cannot fully utilize the different encoding methods and the greater randomness generated by their combination. In order to fully utilize the different characteristics of various encoding levels and the advantages of mixing different encoding methods, this article proposes a PD5H. This method improves the sensitivity, robustness, and resistance to differential attacks of the algorithm by converting the image into a bitstream, cutting it into different lengths, encoding it, and applying different encryption methods.

The main contributions of PD5H proposed in this article are as follows: (1) On the basis of the original five-dimensional chaotic system, it enhances the correlation of each dimension, which can strengthen the chaotic characteristics of the system. (2) A new diffusion method based on stream encryption was proposed, which combines bit-level and base-level diffusion operations, and simultaneously uses four-base DNA and eight-base DNA diffusion operations. By combining multiple encryption operations, a new encryption method with high key sensitivity and good performance was finally obtained. (3) The experiment shows that the proposed PD5H has achieved good results in color image encryption and can effectively resist various types of attacks. The key innovation of this work is the proposal of an encryption algorithm based on hyper chaotic systems with bit-stream encryption as the core.

The main structure of this article is as follows: Section 1 mainly analyzes the current status of DNA encryption methods based on chaotic systems and lists the advanced directions and innovative points of current research. Section 2 briefly introduces the chaotic system referenced and modified in this article and the four-base DNA and eight-base DNA operations. Section 3 elaborates on the specific methods and processes of encryption in detail. Section 4 performs experimental testing of encryption methods to resist various attacks and data comparison with other advanced methods. Section 5 provides a brief summary of the work we have conducted. 

## 2. Preliminaries

### 2.1. Hyperchaotic System

In 2024, Saleh Mobayen et al. proposed a five-dimensional hyperchaotic system, whose algebraic expression is as follows [38]:

(1) $$\left\{\begin{array}{c} \dot{x}=a\left(x+y\right)-k\left(m+3nu^{2}\right)w,\\ \dot{y}=dxz-ex,\\ \dot{z}=rxy-cz,\\ \dot{w}=by-gw,\\ \dot{u}=w. \end{array}\right.$$

In the above equation, *x*, *y*, *z*, *w*, and *u* are state variables, and the system parameters are *a*, *b*, *c*, *d*, *e*, *k*, *r*, *g*, *m*, and *n*. When the initial conditions were set as (*x*, *y*, *z*, *w*, *u*) = (0, 0, 0, 1, 0) and the system parameters were *a* = −10, *b* = 1, *c* = 3, *k* = 1, *d* = 9.8, *e* = 45, *r* = −10, *g* = 5, *m* = 1, and *n* = 2/3, with a time step of 0.01 s and a time length of 100 s, numerical simulations were conducted using Matlab. The corresponding Lyapunov exponents are as follows: *LE1* = 1.2068, *LE2* = 0.002, *LE3* = −0.0032, *LE4* = −5.2070, and *LE5* = −13.9665, with two of them being positive. The sum of the five Lyapunov exponents is negative, indicating that System (1) is a hyperchaotic system.

### 2.2. Four-Base DNA Operation and Eight-Base DNA Operation

Traditional encryption methods, such as data encryption standard (DES) and advanced encryption standard (AES), cannot effectively eliminate images’ local correlation and statistical features. However, DNA operations can effectively eliminate the statistical features of images and reduce their local correlation through complementary and algebraic operations.

The DNA operation in image encryption is a cryptographic method inspired by molecular biology. Its core lies in mapping image data to a symbol system composed of simulated DNA bases, and performing operations based on specific rules to achieve deep confusion and diffusion. Under the condition of ignoring different encoding methods, there are only eight theoretical monocular calculation methods for traditional four-base DNA operations that satisfy the complementarity rule, with a small variation space and easy to be brute-force cracked. Under the same conditions, there are as many as 384 theoretical calculation methods for eight-base DNA operations that satisfy the complementarity rule, greatly expanding the diversity of encryption methods.

However, the eight-base encoding takes a long time and occupies a large space, so we propose a joint diffusion method that combines a four-base DNA operation and an eight-base DNA operation, which can ensure that the encryption effect is not affected while reducing the encoding and decoding time.

#### 2.2.1. Four-Base DNA Operation

There are 24 encoding methods for encoding two bits of an image into DNA bases, but only eight combinations comply with the principle of base complementarity. Here is a list of encoding methods that meet the requirements, as shown in Table 1.

Using different encoding methods for the same image can result in different base sequences. Complementary or algebraic operations are often used for encryption, and here are a few common algebraic operations, as shown in Table 2.

#### 2.2.2. Eight-Base DNA Operation

On the basis of the four-base operation, four new artificial bases were added, which are also paired with each other. Unlike four-base operations, eight-base operations require the use of three bits for encoding, which is very friendly for three-channel images. Each channel can be recombined bit by bit, breaking certain correlations, and there will be no remaining bits generated during processing, allowing for more symmetric encryption.

Here are some coding rules that meet complementary requirements (not all), as shown in Table 3.

Here are some algebraic operations for eight-base DNA, as shown in Table 4, Table 5 and Table 6.

The eight-base DNA manipulation greatly increases the diversity of DNA manipulation, expanding coding and computational methods. Due to the requirement of encoding eight DNA bases using three bits, the encryption of three-channel color images is very friendly and can be widely applied to different encryption methods.

## 3. The Improved Color Image Encryption Approach

### 3.1. Improved Five-Dimensional Chaotic System

In order to improve the performance of System (1), we propose an improved system in the Equation (2):

(2) $$\left\{\begin{array}{c} \dot{x}=a\left(x+y\right)-k\left(m+3nu^{2}\right)w,\\ \dot{y}=dxz-ex,\\ \dot{z}=rxy-cz+zw,\\ \dot{w}=-by-fw,\\ \dot{u}=tx-hw. \end{array}\right.$$

In the above equation, *x*, *y*, *z*, *w*, and *u* are state variables. When the initial conditions were set to (*x*, *y*, *z*, *w*, *u*) = (1, 0, 1, 0, −3.5) and the system parameters were *a* = −10, *b* = 1, *c* = 3, *d* = 9.8, *e* = 45, *f* = 4, *k* = 1, *r* = −10, *m* = 1, *n* = 2/3, *t* = 2, *h* = 0.7, the time step was 0.01 s and the time length was 3000 s, numerical simulations were conducted using Matlab. 

The coupling selection between high-dimensional chaotic systems is an important topic. Excessive coupling can lead to system synchronization, while insufficient coupling can lead to system decoupling, resulting in a decrease in the chaos of chaotic systems. Analyzing the information flow of the original chaotic system, it can be found that there is no direct information flow between y,z and w,u, which may lead to the chaotic behavior of the chaotic system being too simple and even cause the chaotic system to degenerate into a normal system. Therefore, we have appropriately enhanced the information flow of w,u and x,y,z, enhancing the chaotic behavior of the system.

#### 3.1.1. Attractor Phase Diagram

**Figure 1:**
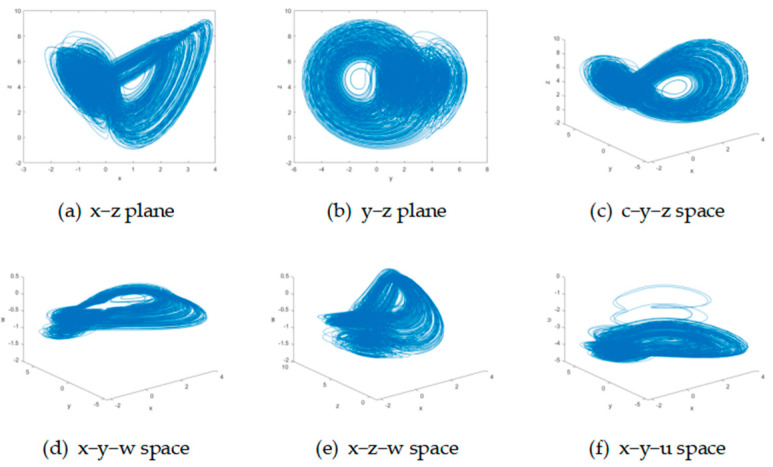
The attractors of the improved five-dimensional chaotic system. The first row shows x−z plane, y−z plane, and x−y−z space, from left to right. The second row shows x−y−w space, x−z−w space and x−y−u space, from left to right.

Here is the attractor phase diagram of the system, as shown in Figure 1.

Compared to the Lorentz-like phase diagram of the original system rules, the improved system exhibits more complex chaotic behavior and better performance.

#### 3.1.2. Lyapunov Exponent

The Lyapunov exponent dimension, commonly referred to as Kaplan–Yorke dimension or Lyapunov dimension, is a method of estimating the fractal dimension of attractors in dynamical systems through the Lyapunov exponent spectrum of the system. The core idea is based on the Kaplan–Yarke conjecture, which links the Lyapunov exponent with geometric dimensions to describe the complex structure of chaotic attractors.

The corresponding Lyapunov exponent is as follows: *LE1* = 1.4002, *LE2* = 0.8084, *LE3* = 0.6665, *LE4* = −0.1335, and *LE5* = −10.5936. It contains three Lyapunov exponents, and the maximum Lyapunov exponent has increased by 16% compared to the original system, showing a significant improvement. The dimension of its Lyapunov exponent is denoted as $D_{KY}$, which is as follows:

(3) $$D_{KY}=4+\frac{LE1+LE2+LE3+LE4}{\left|LE5\right|}=4.2588.$$

The Lyapunov exponent dimension of the original system is denoted as $D_{KY}^{\prime }$, which is as follows:

(4) $$D_{KY}^{\prime }=4+\frac{LE1+LE2+LE3+LE4}{\left|LE5\right|}=3.7135.$$

Compared to the original system, the Lyapunov Exponent dimension has improved by about 0.55. This represents that the system has a divergent trend in more dimensions, so the improved system has better performance.

**Figure 2:**
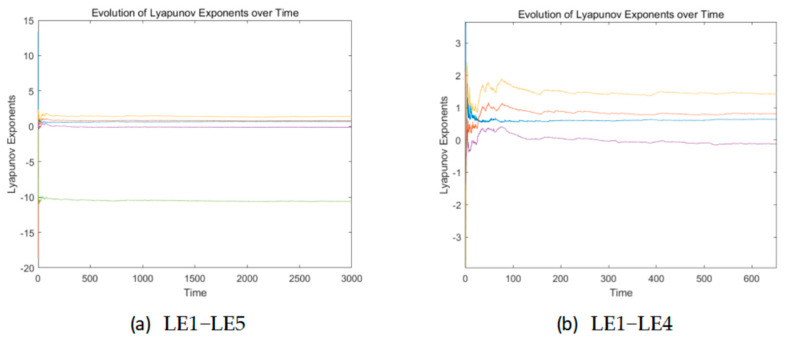
Time-varying exponent spectrum.

Figure 2 shows the time-varying Lyapunov exponent spectrum of the system, which can visually demonstrate the process of Lyapunov exponent tending to stability. The lines from top to bottom in (a) are LE1 to LE5, and the lines from top to bottom in (b) are LE1 to LE4.

#### 3.1.3. Poincaré Map

The Poincaré map simplifies the analysis of long-term dynamical behavior by selecting a hyperplane section in phase space and recording the intersection points of the dynamical system trajectory each time it passes through that section in a unidirectional manner. It transforms continuous-time dynamical systems into discrete maps. The improved Poincaré diagram of the system reveals a complex fractal structure.

**Figure 3:**
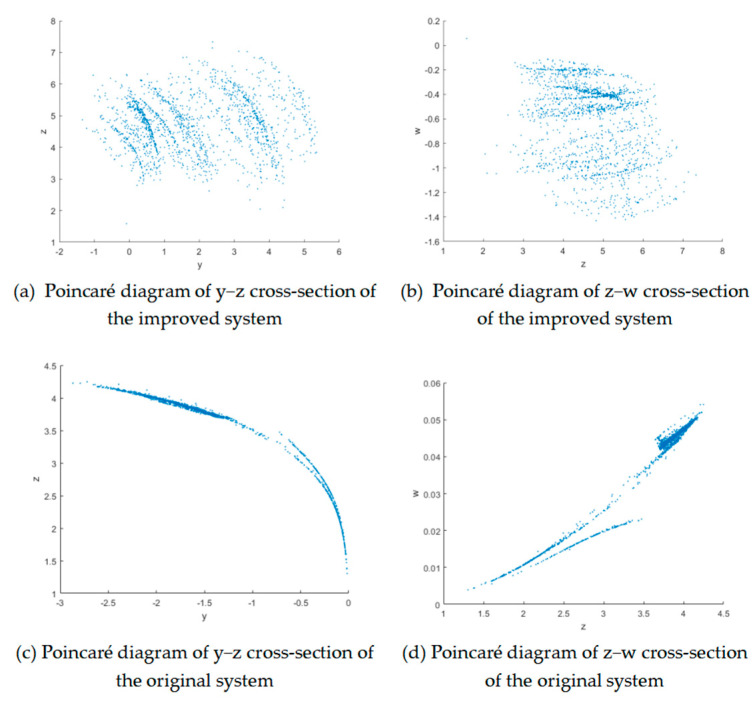
Poincaré diagram of the two systems.

It can be seen that the two Poincaré diagrams of the original system are clearly bounded and clustered, indicating that the original system is a quasi-periodic motion with no obvious chaos from Figure 3. The Poincaré diagram of the improved chaotic system has a complex fractal structure; therefore, the improved chaotic system is more chaotic.

#### 3.1.4. NIST Test 

NIST testing is a statistical testing suite developed by the National Institute of Standards and Technology in the United States to evaluate the quality of random number generators. It includes 15 core tests, such as frequency check, run length check, etc., which detect defects in encryption algorithms or random number generators by analyzing the randomness of binary sequences. This is an important standard for secure authentication of cryptographic products. NIST SP800-22 is a widely accepted suite for verifying the randomness of chaotic sequences [39]. To verify the effectiveness of our improvement, we present the results of the NIST operation for both our chaotic system and the improved chaotic system. The result is as Table 7.

The items marked with “*” in the table represent multiple test results, therefore they are expressed in the form of scores as “number of passed items/total number of tested items”.

From the table, it can be seen that our improved system performs well on the original chaotic system. Although both systems can pass all the tests conducted by NIST, the *p*-value of the improved system is more uniform and higher. Especially in the “w”, “u” two dimensions, the “Rank” and “Cumulative Sums Test” values in the original system are significantly lower compared to other dimensions, which proves that these two dimensions have certain randomness defects. Our improved system has solved this problem to a certain extent.

### 3.2. Generation of Hyperchaotic Sequences

The encryption method of using the same key for multiple different images is susceptible to known plaintext and selective plaintext attacks. To solve this problem, a mainstream method is to generate a key using the information of the image itself, with each image corresponding to its unique key. This enhances the ability to resist known plaintext and selective plaintext attacks. Even if the cracker already knows the correspondence between some plaintext and ciphertext, as well as the encryption method, they cannot quickly crack the ciphertext. We use this method to generate initial values for chaotic systems. Specifically, the initial values of chaotic systems are generated using the following method:

Step 1: The 3D image is flattened into a one-dimensional sequence. The SHA-256 hash of this sequence is computed, resulting in a 256-bit digest. This digest is then split into four contiguous 64-bit blocks and a 64-bit constant C constituting the key set $Key=\{k_{1},k_{2},k_{3},k_{4},C\}$;
Step 2: An intermediate variable $IV=\left\{{iv}_{1},{iv}_{2},{iv}_{3},{iv}_{4}\right\}$ is introduced. For each Key member $k_{i}\;$(where *i* = 1, 2, 3, 4), the following two operations are performed sequentially:
  (5) $$\left\{\begin{array}{c} {iv}_{i}=C⊕k_{i},\\ C\leftarrow rotl\left(C,48\right). \end{array}\right.$$
  where rotl(C,48) denotes the cyclic left shift C by 48 bits. This operation is equivalent to (C≪16)∨(C≫48), where ≪ and ≫ are logical shifts, and ∨ is the bitwise OR operation. Similarly, rotr (C, 48) denotes the cyclic right shift C by 16 bits, equivalent to (C≫16)∨(C≪48).
Step 3: The four 64-bit IV is concatenated into a single 256-bit block, which is then interpreted as a sequence of 32 bytes (8-bit unsigned integers):
  (6) $$B=(b_{1},b_{2},\ldots ,b_{32}).$$
  A new key sequence $KS=\left({ks}_{1},{ks}_{2},\ldots ,{ks}_{16}\right)$ is generated from B to enhance diffusion. For each member of KS, $ks_{\left(j+1\right)\%2}$ (*j =* 1, 3, 5, …, 31) obtains its value using the following formula:
  (7) $$ks_{\left(j+1\right)\%2}=b_{j}⨁b_{j\%32+1}⨁b_{\left(j+1\right)\%32+1}⨁b_{\left(j+2\right)\%32+1}.$$
Step 4: Record the initial value as
  $Init\_Val=\{{initval}_{1},{Iinitval}_{2},{initval}_{3},{initval}_{4},{initval}_{5}\}$ and calculate the initial value as follows:
  (8) $$\left\{\begin{array}{c} {initval}_{1}=\sum_{i=1}^{3}{ks}_{i}/256,\\ {initval}_{2}=\sum_{i=4}^{6}{ks}_{i}/256,\\ {initval}_{3}=\sum_{i=7}^{9}{ks}_{i}/256,\\ {initval}_{4}=\sum_{i=10}^{12}{ks}_{i}/256,\\ {initval}_{5}=\sum_{i=13}^{16}{ks}_{i}/256. \end{array}\right.$$
Step 5: Input the control parameters *a*, *b*, *c*, *d*, *e*, *f*, *g*, *k*, *r*, *m*, *n*, *t,* and *h* with initial values Init_Val into System (2) to generate a hyperchaotic matrix, as S has a size of 5×(w×d×3).

The key can be divided into SHA256 and a custom key part C, so we will discuss it in two parts.

Every change in the SHA part will result in a corresponding change in $b_{x}\;$(scope of x matters 1–32), which in turn will cause a change in two ks. Under pessimistic estimates, it will cause at least one initial value to change, while under optimistic estimates, it will cause at most two initial values to change. In summary, the expected value is 1.625.

Changing one bit of the custom key C will cause four $b_{x}$ to change. In a pessimistic situation, it will cause three initial values to change. In an optimistic situation, it will cause four initial values to change. So key design is effective and can be used to spread small changes in the key.

### 3.3. Padding

In order to lay a solid foundation for intra-block encryption in the future, padding is performed once. In the complement operation, parity verification bits are introduced to smoothly remove the filled pixels during decryption. Filling is performed using the first rows, last columns method, as follows:

Step 1: If the number of columns is even, skip this step and proceed to Step 2. If the number of columns is odd, duplicate the last column and append it to the image.
Step 2: Perform a parity check on the image. Use the last bit of the last column of the image as the parity check bit. This step ensures the number of 1s in the Least Significant Bits (LSBs) of every row is even, effectively using the LSB of the last pixel in each row as a parity bit.
Step 3: If the number of rows is even, end. If the number of rows is odd, duplicate the last row of pixels as a new row of pixels.

In this way, a new image is obtained, whose rows meet the parity check requirements and the number of rows and columns is even, as h and w.

### 3.4. Ascending Sort

Take a hyperchaotic matrix with a size of 3 × (*h* × *w*) from S and interleave the matrix into a one-dimensional sequence, as $S_{1}=\{s_{1}^{1},s_{2}^{1},s_{3}^{1},\ldots ,s_{3\times h\times w}^{1}\}$. Then, adjust the values of the hyperchaotic sequence $S_{1}$ to integers within 0 to 1000 using Equation (9).

(9) $$S_{1}=\left|sin\left({(S}_{1}-\left\lfloor S_{1}\right\rfloor )\times \Pi \right)\right|.$$

Sort $S_{1}$ in ascending order, record the original index of each element during the sorting process, and record the index of the sorting result as $Index=\left[{index}_{1},{index}_{2},{index}_{3},\ldots ,{index}_{3\times h\times w}\right]$. Let the filled image be P, interleave P into a one-dimensional integer sequence, denoted as $PL=\left[{pl}_{1},{pl}_{2},{pl}_{3},\ldots ,{pl}_{3\times h\times w}\right]$, and then perform the following operation on all members ${pl}_{i}$ of PL:

(10) $${pl}_{i}={pl}_{{index}_{i}}.$$

**Figure 4:**
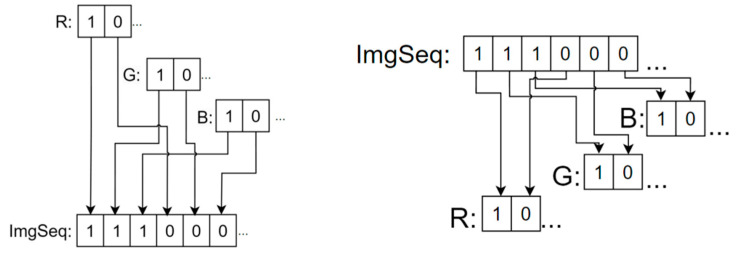
The “interleave” (**left**) and “deinterleave” (**right**).

The “interleave” is shown in Figure 4. The “interleave” (left) and “deinterleave” (right).

Then, convert PL back into a 3D image.

### 3.5. Intra-Block Diffusion

Adopting a divide and conquer strategy, the image is segmented into 2 × 2 pixel blocks, and dynamic address mapping is used as the basis for intra-block encryption. Intra-block diffusion is then performed on each pixel block.

Using the following method, first expand the pixel block, mark them as ${PB}_{1}$–${PB}_{4}$ in order, mark the current pixel as ${PB}_{i}$, and then confirm the previous pixel ${PB}_{j}$ in the following way:

(11) $$j=\left\{\begin{array}{c} i-1\left(if\;i>=2\right).\\ 4\left(if\;i==1\right). \end{array}\right.$$

Step 1: Determine two mapping addresses as follows:
  (12) $$\left\{\begin{array}{c} {Add}_{1}={PB}_{i}⊕7+1,\\ {Add}_{2}=\left\lfloor \left({PB}_{i}⊕72\right)\%8\right\rfloor +1. \end{array}\right.$$
Step 2: Mark each bit ${PB}_{i}[m]$ of ${PB}_{i}$ and perform the following operation:
  (13) $${PB}_{i}[m]\leftarrow \left\{\begin{array}{c} {PB}_{i}\left[m\right].(if\;m=={Add}_{1}\;or\;{Add}_{2})\\ \left\{\begin{array}{c} {PB}_{i}\left[m\right]⊕{PB}_{i}\left[{Add}_{1}\right]\left(m\%2==0\right).\\ {PB}_{i}\left[m\right]⊕{PB}_{i}\left[{Add}_{2}\right]\left(m\%2==1\right). \end{array}(if\;m!={Add}_{1}\;or\;{Add}_{2})\right. \end{array}\right.$$
Step 3: Exchange the first three bits of ${PB}_{i}$ and the last three bits of ${PB}_{j}$. This operation enhances the strength of diffusion and eliminates the possibility of both dynamic mapping bits having a value of 0, resulting in an unsatisfactory XOR result. At the same time, this operation can prevent the scenario where both mapping addresses point to bits with value 0, which could reduce the effectiveness of XOR operations.

**Figure 5:**
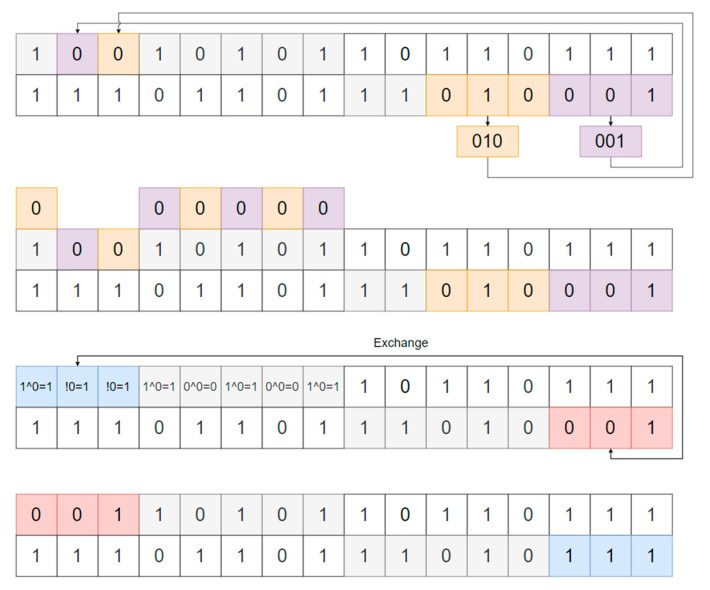
Schematic diagram of one round of intra-block encryption. ^⋀^ represents XOR, and ! represents opposition.

The index i is iterated from one to four, and for each pixel ${PB}_{i}$ in the block. Step 1, Step 2, and Step 3 are repeated and exchanged to complete the encryption within the entire block. Figure 5 is a schematic diagram of the intra-block diffusion.

### 3.6. Joint Diffusion

Convert the remaining chaotic matrix into a one-dimensional sequence, denoted as $S_{2}=\left\{s_{1}^{2},s_{2}^{2},s_{3}^{2},\ldots \right\}$, using Equation (14) to convert it into an eight-bit unsigned integer, denoted as $ConSeq=\left\{{conseqs}_{1},{sconseq}_{2},{conseq}_{3},\ldots \right\}$ with a length of lengthc, and then perform the following operation on all members ${conseqs}_{i}$ of ConSeq, which $s_{i}^{2}$ is a member of $S_{2}$:

(14) $${conseq}_{i}=\left\lfloor \left(s_{i}^{2}\times 10^{5}\right)\%256\right\rfloor .$$

Set the two flag bits, Flagi and Flagc, to 1, pointing to the first bit of ImgSeq and ConSeq, respectively. The length of Flagi is denoted as lengthi. And set the result array to ResSeq. Next, proceed with the following steps:

Step 1: If the size of Flagi is greater than or equal to lengthi, then end the entire algorithm. If the difference between Flagi and lengthi is not greater than *4*, record the difference as Casen, execute $Case\left[Casen\right]$, and then immediately end the algorithm. If it is greater, then take ConSeq[Flagc] and ConSeq[Flagc+1] (hereinafter referred to as ConSeq[Flagc:Flagc+1]) to be the same as ImgSeq.
Step 2: Convert ConSeq[Flagc:Flagc+1] as a binary number to an integer X and execute Case[X] and let
  (15) Flagc=Flagc+2.
  Case [0]: XOR ConSeq[Flagc] with ImgSeq[Flagi], as
    (16) $$\left\{\begin{array}{c} ResSeq\left[Flagi\right]=ConSeq\left[Flagc\right]⊕ImgSeq\left[Flagi\right],\\ Flagc=Flagc+1,\\ Flagi=Flagi+1. \end{array}\right.$$
    where ⊕ refers to the bitwise XOR operation.
  Case [1]: Take ConSeq[Flagc:Flagc+1] and convert it into DNA base X1 according to rule 1 in Table 1, take ImgSeq [Flagi: Flagi+1] and convert it into a DNA base according to rule 1 in Table 1, denoted as X2. Let
    (17) $$\left\{\begin{array}{c} ResSeq\left[Flagi:Flagi+1\right]=DNADecode\left(X1⊕X2\right),\\ Flagc=Flagc+2,\\ Flagi=Flagi+2. \end{array}\right.$$
    where ⊕ denotes the base XOR operation, performed according to the rules specified in Table 2, and DNADecode() represents the function that converts bases back into their binary bit sequences according to rule 1 in Table 1.
  Case [2]: Take ConSeq[Flagc: Flagc+2] and convert it into an eight-base DNA base according to rule 1 in Table 3. Eight-Base DNA encoding rules., denoted as X3. Take ImgSeq[Flagi: Flagi+2] and convert it into an eight-base DNA base according to rule 1 in Table 3. Eight-Base DNA encoding rules., denoted as X4. Let
    (18) $$\left\{\begin{array}{c} ResSeq\left[Flagi:Flagi+2\right]=DNADecode\left(X3⊕X4\right),\\ Flagc=Flagc+3,\\ Flagi=Flagi+3. \end{array}\right.$$
    where ⊕ refers to base XOR operation, which is performed according to the method specified in Table 3. Eight-Base DNA encoding rules., and DNADecode() represents the function that converts bases back into their binary bit sequences according to rule 1 in Table 3. Eight-Base DNA encoding rules.
  Case [3]: Take ConSeq[Flagc: Flagc+1], denoted as Y, and perform a new classification encryption according to the following method. For ease of expression, the form of ‘Casem []’ is used to distinguish, and execute Equation (15).
    Then, execute Casem [Y].
    Casem [0]: Take ImgSeq[Flagi: Flagi+3], denoted as N, invert N bit by bit, denoted as $\bar{N}$, and let
      (19) $$\left\{\begin{array}{c} ResSeq\left[Flagi:Flagi+3\right]=\bar{N},\\ Flagi=Flagi+4. \end{array}\right.$$
    Casem [1]: Take ConSeq[Flagc: Flagc+1] , denoted as integer K, and let
      (20) $$\left\{\begin{array}{c} ResSeq\left[Flagi:Flagi+3\right]=rotr\left(ImgSeq\left[Flagi:Flagi+3\right],K\right),\\ Flagi=Flagi+4,\\ Flagc=Flagc+2. \end{array}\right.$$
    Casem [2]: Take $ConSeq\left[Flagc:Flagc+3\right]$, denoted as integer K, and let
      (21) $$\left\{\begin{array}{c} ResSeq\left[Flagi:Flagi+3\right]=rotl\left(ImgSeq\left[Flagi:Flagi+3\right],K\right),\\ Flagi=Flagi+4,\\ Flagc=Flagc+2. \end{array}\right.$$
    Casem [3]: Take ImgSeq[Flagi:Flagi+3], and take ConSeq[Flagc:Flagc+3], and let

(22) $$\left\{\begin{array}{c} ResSeq\left[Flagi:Flagi+3\right]=ConSeq\left[Flagc:Flagc+3\right]⊕ImgSeq\left[Flagi:Flagi+3\right],\\ Flagi=Flagi+4,\\ Flagc=Flagc+4. \end{array}\right.$$

where ⊕ refers to the bitwise XOR operation.

Repeat Step 1 and Step 2 until the flagi exceeds the lengthi.

After executing all processes, a one-dimensional bit sequence with a size equal to ImgSeq will be obtained. For each case analysis, encrypting 1 bit of ImgSeq on average requires 2.05 ConSeq bits, with a maximum of 3 required. However, for the stability of the encryption algorithm, a ConSeq sequence with three times the length of ImgSeq is used to ensure complete processing of all ImgSeqs.

This process is the core process of this encryption, which encrypts sequences of different lengths through a variable window. Therefore, any change in the sequence at any position will result in completely different encryption results. Additionally, due to the randomness and extreme sensitivity of the hyper chaotic system to initial values, combined with the different keys generated by SHA-256 for each image, it can be guaranteed that even a slight change will lead to completely different encryption effects.

Due to the slow speed of streaming encryption, a multi-threaded approach can be adopted to improve the encryption speed. Divide ImgSeq and ConSeq into n equal parts to perform n sets of encryptions simultaneously. After actual testing, under the experimental conditions of this paper, the encryption speed is the fastest when n=16.

### 3.7. Image Recombination

The one-dimensional bit sequence obtained by joint diffusion is recombined and restored into a three-channel color image by bit deinterleaving to obtain the final encrypted image. Figure 4 has a schematic diagram of deinterleaving in bits.

### 3.8. Encryption Method Framework of the Proposed PD5H

Step 1: Key generation: Generate initial values using SHA-256 hash values and custom constants, and combine them with control parameters to construct a chaotic system.
Step 2: Padding: Fill the image to an even number of rows and columns, making it easier for subsequent block encryption.
Step 3: Ascending Sort: Unfold the pixels into a one-dimensional sequence and scramble them in ascending order using a chaotic sequence.
Step 4: Intra-block diffusion: Divide the image into 2 × 2 pixel blocks and use dynamic address mapping for intra-block encryption.
Step 5: Joint diffusion: Convert the image and chaotic sequence into a one-dimensional bit sequence, and perform flow diffusion by combining four-base and eight-base DNA operations.
Step 6: Image Recombination: Recombine the encrypted bit sequence into a three-dimensional color image.

**Figure 6:**
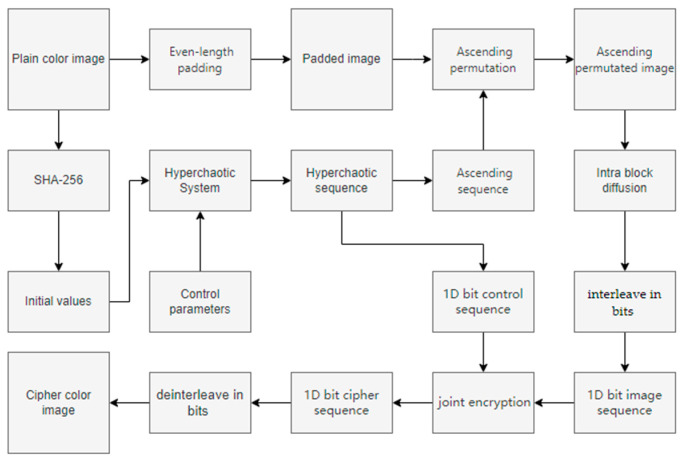
Schematic diagram of the encryption process.

The schematic diagram of the encryption process is shown in Figure 6.

## 4. Experimental Results

### 4.1. Experimental Setup

The performance of the proposed PD5H was evaluated through a series of experiments and compared against other image encryption methods. The number of iterations for generating the hyper chaotic sequence was set to h × d × 3, ensuring the sequence length is sufficient for encryption, where h and d represent the height and width of the original image, respectively. The following test image sizes were used as shown in Table 8 in our experiments:

All experiments were conducted using MATLAB R2023a (Mathworks, Natick, MA, USA) on a PC with a 64-bit Windows 11 operating system (Microsoft, Redmond, WA, USA), a 2.40 GHz R9-7940HX CPU, and 32 GB RAM.

### 4.2. Key Analysis

The cryptographic key is a critical component for the security of any encryption method. To be considered secure, a key must exhibit two essential characteristics: a sufficiently large key space to prevent brute-force attacks and high key sensitivity to ensure that similar keys produce vastly different ciphertexts.

#### 4.2.1. Key Space Analysis

A sufficiently large key space can effectively resist brute-force attacks. Research has shown that a key space larger than $2^{128}$ can effectively resist brute-force attacks. The key in this article consists of two parts: the SHA256 value of the image and a custom constant C. The length of the key is 256+64=320, which should have a large key space of $2^{320}$, but as we consider the issue of equivalent keys, the key space has decreased to${\;2}^{256}$. It is still much larger than $2^{128}$, so it can effectively resist violent attacks. Meanwhile, our key is composed of two different parts, greatly enhancing the algorithm’s resistance to brute-force cryptanalysis.

#### 4.2.2. Key Sensitivity Analysis

Key sensitivity is a fundamental security criterion for encryption algorithms, measuring the effect of minute key alterations on the corresponding ciphertext. In a key-sensitive system, even a single-bit change in the key should produce a completely different ciphertext when applied to the same plaintext, thereby preventing statistical analysis or key deduction based on similar encryption outcomes. 

The key used in this article is divided into two parts, and there are significant differences between them. Therefore, we divided the testing into two experiments.

This article uses SHA-256 to generate the key corresponding to each image, so another sensitive method is adopted, which is to use a new key, ${Key}_{2}$ that is one bit different from the original key, ${Key}_{1},\;$for the cipher image, which is as follows:

(23) $${Key}_{2}={Key}_{1}⊕1.$$

**Figure 7:**
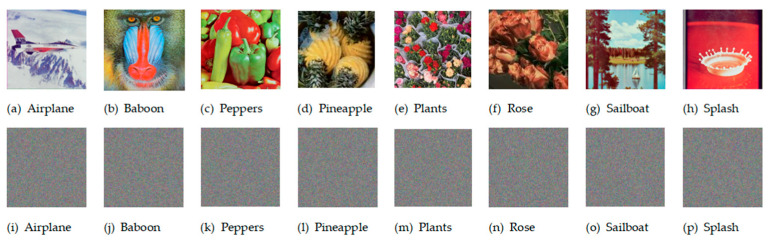
Original image (**top**) and decrypted image (**bottom**).

The ciphertext encrypted with the original key is then decrypted using this slightly modified ${Key}_{2}$ to evaluate the sensitivity of the decryption process to minute key alterations, as shown in the Figure 7.

NPCR (Number of Pixels Change Rate) is a quantitative measure used to evaluate the sensitivity of an encryption algorithm. It represents the proportion of changed pixels to the total number of pixels after encryption, under a minimal change in the key or plaintext. The calculation is given by Equation (24):

(24) $$NCPR=\frac{1}{h\times w}\sum_{i=1}^{h}\sum_{j=1}^{w}\delta_{i,j}\times 100\%,$$

where $\delta_{i,j}$ is the equal relationship between the pixels at the positions of two images (*i*, *j*), where 0 represents the same, and 1 represents different.

UACI (Unified Average Changing Intensity) measures the average intensity difference between corresponding pixels in two encrypted images. It serves as a standard benchmark for evaluating the effectiveness of an encryption algorithm, with a widely referenced ideal value of approximately 33.4%. The calculation is given by Equation (25):

(25) $$UACI=\frac{1}{h\times w\times 255}\sum_{i=1}^{h}\sum_{j=1}^{w}\left|Q_{i,j}^{1}-Q_{i,j}^{2}\right|\times 100\%.$$

where $Q_{i,j}^{1}$ X is the pixel value at position (*i*, *j*) in image 1.

We change the custom constant C by one bit and then construct a new key to record different encryption results. The results are shown in rows 3 and 4 of Table 9.

Taking into account the key sensitivity of both parts, we can draw the following conclusion: even when only a single bit of the key is altered, the decrypted image becomes entirely unrecognizable. The NPCR value approaches the ideal benchmark of 99.6%, while the UACI, though slightly below its ideal value, remains sufficiently high. These results demonstrate that the PD5H algorithm exhibits strong key sensitivity. From the attacker’s perspective, the actual performance is lower than the highest value, but it can still resist most attacks.

### 4.3. Statistical Analysis

In the field of image encryption, statistical analysis refers to the use of mathematical tools to evaluate the statistical properties of encrypted images in order to determine whether they can effectively conceal the statistical patterns of the original image. A robust encryption method must demonstrate strong resistance against statistical analysis, including attacks based on histogram analysis, information entropy analysis, and correlation analysis.

#### 4.3.1. Histogram Analysis

**Figure 8:**
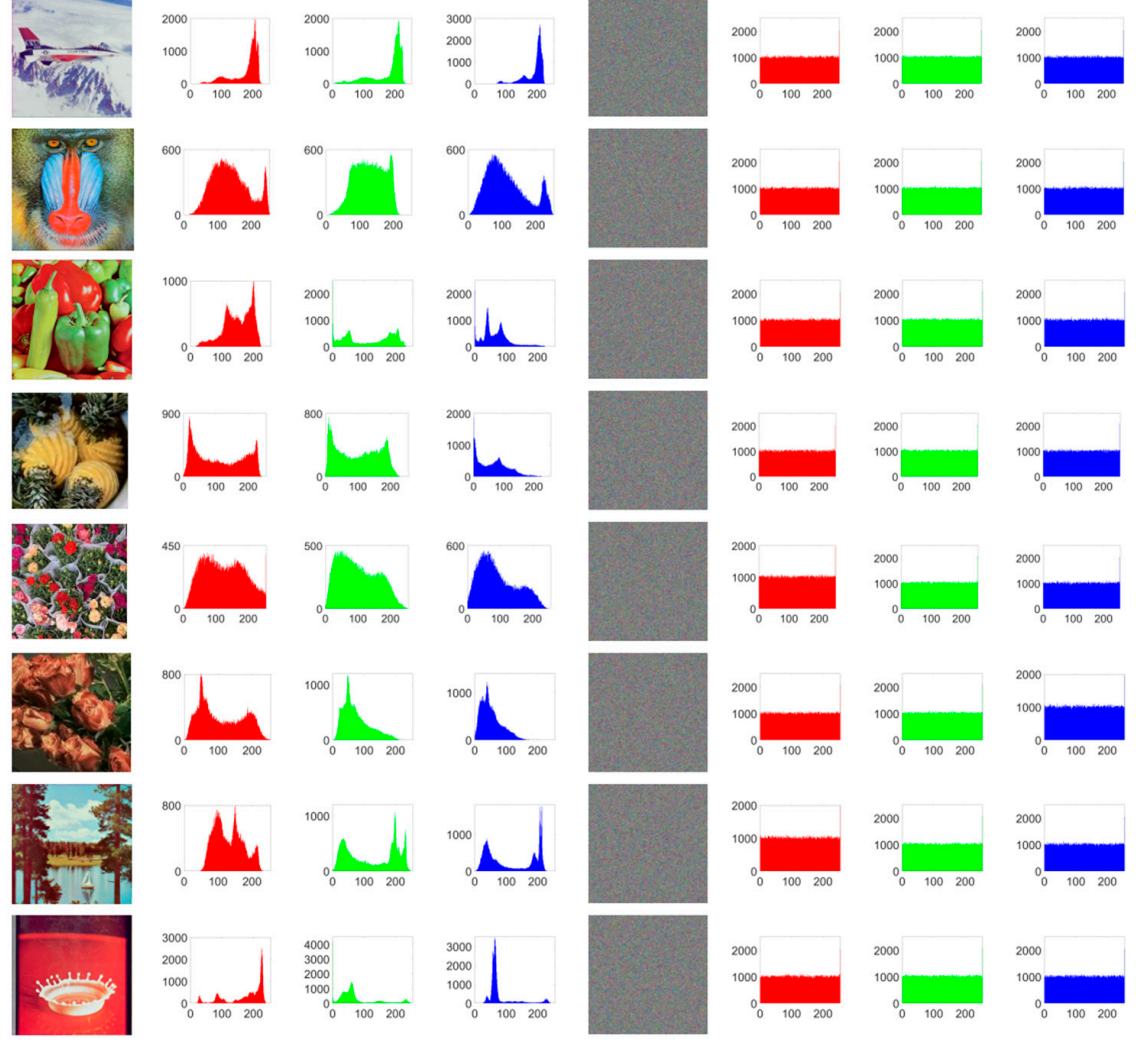
Original image (column 1) and its Red, Green and Blue histogram (columns 2–4) and encrypted image(column 5) and its Red, Green and Blue histogram (columns 6–8).

Histogram analysis characterizes an image by counting the frequency of each pixel value, thereby revealing its statistical features. Attackers often use histogram analysis to infer encryption methods or deduce key information. An effectively encrypted image should exhibit a fairly uniform histogram to better conceal the features of the original image Figure 8 shows the original image, the encrypted image, and their respective histograms.

As can be observed from Figure 8, the histograms of different original images exhibit distinct and strong features, whereas the histograms of each channel in the encrypted image are nearly uniform, making it difficult for attackers to extract meaningful information from them. It can be seen that PD5H can effectively resist histogram attacks.

#### 4.3.2. Information Entropy

In 1948, American scientist Claude E. Shannon introduced the concept of information entropy as a mathematical measure of uncertainty in information. This metric has since been extensively adopted in image processing. For an encrypted image, a desirable information entropy value should be close to eight, reflecting a high degree of randomness resembling high-frequency noise. 

For channel C with a gray level of $2^{8}=256,$ $p_{i}$ is the probability of a pixel with a gray value of i appearing, and its information entropy (*EI*(*C*)) can be calculated by Equation (26).

(26) $$EI\left(C\right)=-\sum_{i=0}^{255}p_{i}{log}_{2}p_{i}.$$

Table 9 presents a comparison of information entropy between original and encrypted images using PD5H and several other encryption methods.

As shown in the table, the information entropy of original images typically ranges between 6.5 and 7.5. Among these, the image “Plants” exhibits relatively high entropy owing to its rich detail. In contrast, the encrypted images consistently achieve information entropy values above 7.999 in each channel, indicating that the cipher images approximate the statistical characteristics of high-frequency noise and are difficult to distinguish from random data.

When compared with five other encryption methods, PD5H achieved the highest information entropy in a total of six test cases, demonstrating its superior encryption performance.

#### 4.3.3. Correlation Analysis

Correlation analysis evaluates the degree of association between adjacent pixels in an image. To measure the correlation between pixels in an image in various directions, the correlation coefficient *γ* is defined by Equation (27):

(27) $$E\left(x\right)=\frac{1}{M}\sum_{i=1}^{M}x_{i}\;,\;C=\frac{1}{M}\sum_{i=1}^{M}{\left(x_{i}-E\left(x\right)\right)}^{2},\;cov\left(x,y\right)=\sum_{i=1}^{M}\left(x_{i}-E\left(x\right))(y_{i}-E\left(y\right)\right),\;\gamma =\frac{cov\left(x,y\right)}{\sqrt{E\left(x\right)E\left(y\right)}}\;.$$

In this equation, x and y are the gray levels of two adjacent pixels in the auxiliary image channel; M is the total number of pixel pairs; and E (x), D (x), and cov (x, y) represent the expected value of x, the standard deviation of x, and the covariance of x and y.

In natural images, pixels typically exhibit strong correlations due to structural and semantic continuity. Effective encryption, however, should significantly reduce these correlations, making the encrypted image resemble random noise Table 10 displays the correlation coefficients for each channel of both the original and encrypted images.

The bolded items indicate that different encryption methods exhibit the highest information entropy in the same image and channel.

The original image shows high correlation, with pixel values clustering closely along a diagonal line in a scatter plot. In contrast, the encrypted image exhibits a scattered and disordered distribution, indicating drastically reduced inter-pixel dependency. The correlation coefficients across different channels in the encrypted image are all below *0.005*, demonstrating the absence of significant linear relationships within or across color dimensions.

**Figure 9:**
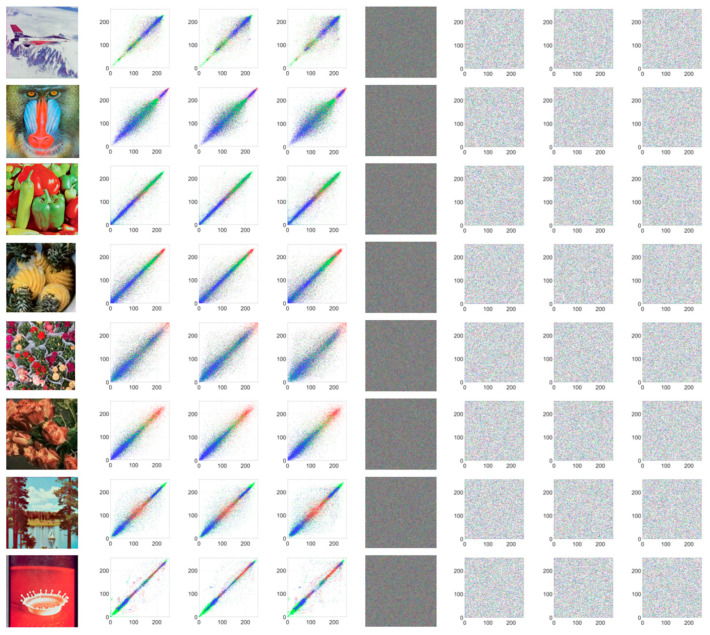
The first column is the original image, the second to fourth columns are the horizontal, vertical, and diagonal pixel correlation statistics of the original image. The fifth column is the encrypted image, and the sixth to eighth columns are the horizontal, vertical, and diagonal pixel correlation statistics of the encrypted image.

A total of 45 correlation measurements—covering all three channels and multiple directional relationships across five test images—were analyzed. Among these, PD5H achieved the lowest correlation values in 16 cases, outperforming the best results from other encryption methods in 14 comparisons. These results as shown in Figure 9 and Table 11 confirm that PD5H effectively disrupts pixel correlations and enhances security against statistical attacks.

Bold items indicate that different encryption methods exhibit the lowest correlation in the same image and channel in Table 11.

### 4.4. Differential Attack Analysis

Differential cryptanalysis is a common attack method in which an adversary encrypts two plaintext images with minimal differences and compares the resulting ciphertexts to deduce information about the encryption algorithm or the key. An encryption scheme is considered vulnerable if small changes in the input image lead to only minor changes in the ciphertext. Conversely, a secure encryption method should produce significantly different ciphertexts even when the plaintexts differ by only one pixel.

To evaluate resistance against differential attacks, a one-bit change was introduced into a single pixel of the original image. The modified image was then encrypted, and the resulting ciphertext was compared with the ciphertext of the unmodified original. The NPCR and UACI values were calculated and are presented in Table 12 and Table 13.

The results show that even after only a one-bit alteration in the plaintext, the NPCR and UACI values of the ciphertext are close to their ideal theoretical values. This indicates that the encryption method produces drastically different outputs for minimally different inputs, demonstrating strong resistance against differential cryptanalysis.

### 4.5. Robustness Analysis

During the storage and transmission of digital images, data corruption and noise interference are inevitable challenges. A robust encryption algorithm should, therefore, maintain both security and decipherability under such adverse conditions, demonstrating resistance to both noise contamination and data loss. This capability ensures the encrypted content remains usable even after common transmission errors or storage defects.

**Figure 10:**
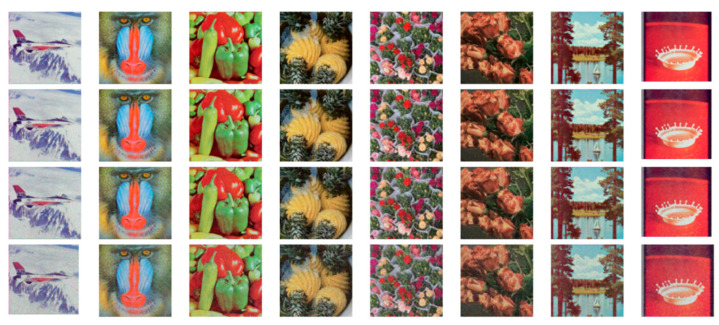
The restoration results of the encrypted image with added noise, with 1%, 2.5%, 5%, and 10% salt and pepper noise added from top to bottom.

Data storage and transmission are susceptible to media damage, which may result in noise. We introduce different proportions of salt and pepper noise into encrypted images and observe their decryption effect. The decrypted images with 1%, 2.5%, 5%, and 10% salt and pepper noise added are shown in Figure 10.

As can be seen, the more noise added, the more noise in the image, but it does not affect the subject of the image. Therefore, PD5H has a strong ability to resist noise.

**Figure 11:**
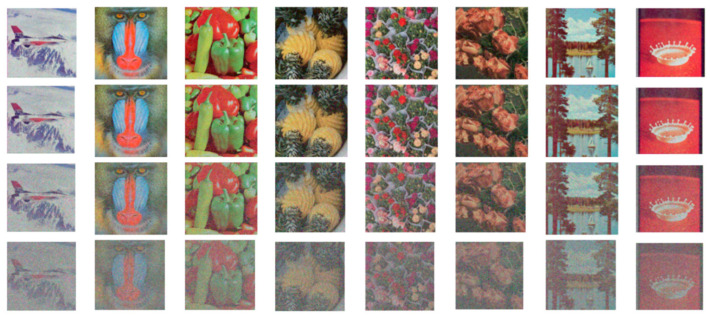
The restoration results of partially cropped encrypted images, with cropping rates of 6.25%, 12.5%, 25%, and 50% from top to bottom.

**Figure 12:**
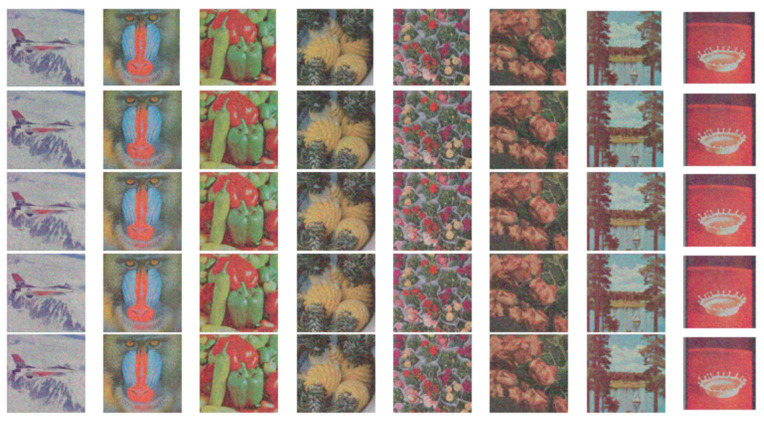
Image reconstruction performance under 25% cropping attack: analysis of top-left, top-right, central, bottom-left, and bottom-right scenarios.

Figure 11 are images decrypted from encrypted images with losses of 6.25%, 12.5%, 25%, and 50%. In order to investigate the impact of cutting attacks at different positions on the robustness of encryption algorithms, we cut the image by 25% at different positions and restored it. The results are shown in Figure 12. It can be seen that even if the encrypted image loses 50%, the restored image still has some practical value and can still distinguish the main content. Different cutting positions will not cause significant changes in the quality of decrypted images. So, PD5H has a high ability to resist pruning attacks.

### 4.6. Time Analysis

The encryption time measures the efficiency of the algorithm and affects the feasibility of real-time applications. The decryption time directly determines the recovery speed of data availability. The two together reflect the performance and practicality of encryption schemes in practical deployment, and are key indicators for measuring their comprehensive value.

Table 14 is the average time spent encrypting and decrypting images of different sizes 64 times using PD5H.

Due to the inability of stream cipher encryption to optimize encryption time using matrix, multithreading, and other methods, the overall encryption speed is slow but still within an acceptable time range.

## 5. Conclusions

This study presents an improved five-dimensional chaotic system characterized by a greater number of positive Lyapunov exponents, a higher maximum Lyapunov exponent, and an increased Lyapunov exponent dimension. These index improvements demonstrate that the chaotic performance of the improved system has significantly improved compared to the original system.

A novel image encryption scheme is proposed based on this system, incorporating several innovative features: a new padding method for encrypted image, a block-based encryption method that utilizes the intrinsic information of pixel blocks, and a joint diffusion mechanism that operates on a bit-level basis by integrating four-base and eight-base DNA operations after integer shaping of the image into a bitstream. 

PD5H has a large key space, extremely low image correlation, a uniform ciphertext pixel distribution, an excellent ciphertext entropy value (>7.999), and strong resistance to differential attacks. It also demonstrates strong resistance to data loss. This approach demonstrates excellent performance, particularly in resisting differential and statistical attacks. However, this encryption scheme has certain limitations: the encryption time is relatively prolonged, and key sensitivity remains slightly inadequate. 

Due to the unique characteristics of stream encryption, PD5H has inherent advantages in terms of software and hardware equivalence and can be applied to low computing power platforms. In addition, PD5H may also be applied in the field of communication, which has reference significance for the transmission and encryption of various data carriers, such as images, videos, audio, etc., making encryption algorithms not limited to mathematical research, but truly applied in communication, storage, and other fields, achieving a wider range of applications.

## Figures and Tables

**Table 1:** Traditional DNA encoding rules.

	Rule 1	Rule 2	Rule 3	Rule 4	Rule 5	Rule 6	Rule 7	Rule 8
A	00	00	11	11	01	10	01	10
T	11	11	00	00	10	01	10	01
C	01	10	01	10	00	00	11	11
G	10	01	10	01	11	11	00	00

**Table 2:** Traditional DNA algebraic addition (⊕), subtraction (⊖), and XOR (⊗) operations.

⊕	A	C	G	T	⊖	A	C	G	T	⊗	A	C	G	T
A	A	C	G	T	A	A	T	G	C	A	A	C	G	T
C	C	G	T	A	C	C	A	T	G	C	C	A	T	G
G	G	T	A	C	G	G	C	A	T	G	G	T	A	C
T	T	A	C	G	T	T	G	C	A	T	T	G	C	A

**Table 3:** Eight-Base DNA encoding rules.

	Rule 1	Rule 2	Rule 3	Rule 4	Rule 5	Rule 6	Rule 7	Rule 8
A	000	111	110	110	000	111	110	110
T	001	110	000	111	001	110	000	111
C	110	001	111	000	110	001	111	000
G	111	000	001	001	111	000	001	001
S	010	101	010	101	101	010	101	010
P	100	011	100	011	011	100	011	100
Z	011	100	011	100	100	011	100	011
B	101	010	101	010	010	101	010	101

**Table 4:** Eight-base DNA algebraic addition (⊕) operations.

⊕	A	Z	C	S	B	G	P	T
A	A	Z	C	S	B	G	P	T
Z	Z	C	S	B	G	P	T	A
C	C	S	B	G	P	T	A	Z
S	S	B	G	P	T	A	Z	C
B	B	G	P	T	A	Z	C	S
G	G	P	T	A	Z	C	S	B
P	P	T	A	Z	C	S	B	G
T	T	A	Z	C	S	B	G	P

**Table 5:** Eight-base DNA algebraic subtraction (⊖) operations.

⊖	A	Z	C	S	B	G	P	T
A	A	T	P	G	B	S	C	Z
Z	Z	A	T	P	G	B	S	C
C	C	Z	A	T	P	G	B	S
S	S	C	Z	A	T	P	G	B
B	B	S	C	Z	A	T	P	G
G	G	B	S	C	Z	A	T	P
P	P	G	B	S	C	Z	A	T
T	T	P	G	B	S	C	Z	A

**Table 6:** Eight-base DNA algebraic XOR (⊗) operations.

⊗	A	Z	C	S	B	G	P	T
A	A	Z	C	S	B	G	P	T
Z	Z	A	S	C	G	B	T	P
C	C	S	A	Z	P	T	B	G
S	S	C	Z	A	T	P	G	B
B	B	G	P	T	A	Z	C	S
G	G	B	T	P	Z	A	S	C
P	P	T	B	G	C	S	A	Z
T	T	P	G	B	S	C	Z	A

**Table 7:** Comparison of NIST SP800-22 test results between initial and improved systems.

		Approximate Entropy	Block Frequency	Rank	FFT	Frequency	Linear Complexity	Longest Run	NonOverlappingTemplate *
Improved System (2)	x	0.205183	0.284290	0.727528	0.926884	0.359667	0.206841	0.760482	148/148
	y	0.609539	0.759712	0.960315	0.025753	0.017597	0.212360	0.423187	147/148
	z	0.823422	0.753399	0.333555	0.905041	0.046152	0.715606	0.651096	148/148
	w	0.839681	0.702103	0.731073	0.358795	0.571394	0.208506	0.935493	148/148
	u	0.889604	0.804598	0.833186	0.713570	0.923521	0.860653	0.206465	148/148
Original System (1)	x	0.348987	0.463629	0.527743	0.679644	0.888660	0.371212	0.254461	148/148
	y	0.613821	0.778943	0.489035	0.588217	0.844610	0.858043	0.992015	148/148
	z	0.537671	0.393689	0.229399	0.075032	0.393105	0.246631	0.397009	148/148
	w	0.067219	0.784958	0.047862	0.330691	0.801041	0.974213	0.227209	148/148
	u	0.399338	0.900969	0.089195	0.087849	0.233261	0.323436	0.411514	148/148
		**OverlappingTemplate**	Random Excursions *	Random Excursions Variant *	**Cumulative Sums**	**Runs**	**Serial**	**UniSerial**	
Improved System (2)	x	0.760728	8/8	18/18	0.418624	0.193313	0.271557	0.468685	
					0.698899		0.542017		
	y	0.087256	8/8	18/18	0.034158	0.799776	0.930077	0.548464	
					0.017127		0.518387		
	z	0.060295	8/8	18/18	0.08451	0.921913	0.839519	0.930918	
					0.027326		0.940196		
	w	0.459411	8/8	18/18	0.104032	0.403335	0.080243	0.335151	
					0.336952		0.134724		
	u	0.490488	8/8	18/18	0.866193	0.230913	0.407834	0.339055	
					0.782085		0.033787		
Original System (1)	x	0.316237	8/8	18/18	0.93133	0.638369	0.651158	0.988522	
					0.988909		0.723165		
	y	0.643462	8/8	18/18	0.932558	0.855613	0.885978	0.716916	
					0.915089		0.529277		
	z	0.043315	8/8	18/18	0.212186	0.019768	0.538232	0.902510	
					0.622832		0.838676		
	w	0.469574	8/8	18/18	0.421519	0.651315	0.257200	0.277518	
					0.265665		0.026861		
	u	0.574529	8/8	18/18	0.328391	0.606258	0.186624	0.387902	
					0.277729		0.731862		

**Table 8:** The sizes of test images.

Image	Size (h × w × d)
Airplane	512 × 512 × 3
Baboon	512 × 512 × 3
Peppers	512 × 512 × 3
Sailboat	512 × 512 × 3
Splash	512 × 512 × 3
Pineapple	512 × 512 × 3
Rose	512 × 512 × 3
Plants	512 × 512 × 3

**Table 9:** The first and second lines are the NCPR and UACI comparison between decrypted images using ${Key}_{2}$ and original images, while the third and fourth lines compare the NCPR and UACI between encrypted images using *C*-values that differ by one bit.

	Airplane	Baboon	Peppers	Pineapple	Plants	Rose	Sailboat	Splash
NPCR	99.61%	99.61%	99.61%	99.61%	99.61%	99.61%	99.61%	99.60%
UACI	32.60%	32.91%	32.20%	33.72%	31.61%	33.17%	32.20%	34.05%
NPCR1	99.60%	99.62%	99.62%	99.62%	99.61%	99.61%	99.61%	99.61%
UACI1	33.49%	33.52%	33.49%	33.49%	33.46%	33.46%	33.44%	33.44%

**Table 10:** Image Information Entropy.

Image	Channel	Input	Cipher Images				
			PD5H	Ref. [20]	Ref. [24]	Ref. [26]	Ref. [32]
Airplane	R	6.7178	7.9993	7.9993	7.9993	**7.9994**	7.9993
	G	6.7990	7.9992	**7.9993**	**7.9993**	**7.9993**	**7.9993**
	B	6.2138	**7.9994**	7.9994	7.9992	7.9993	7.9993
Baboon	R	7.7067	7.9993	7.9993	7.9992	**7.9994**	7.9993
	G	7.4744	7.9994	7.9992	**7.9995**	7.9994	7.9993
	B	7.7522	7.9992	**7.9994**	7.9993	7.9992	7.9993
Peppers	R	7.3388	7.9993	7.9992	7.9992	**7.9994**	**7.9994**
	G	7.4963	**7.9994**	7.9993	7.9993	7.9993	7.9993
	B	7.0583	7.9992	**7.9994**	**7.9994**	7.9993	7.9993
Sailboat	R	7.7570	7.9992	7.9993	7.9992	**7.9994**	7.9993
	G	7.6830	**7.9993**	**7.9993**	**7.9993**	7.9992	**7.9993**
	B	7.2726	7.9993	7.9992	7.9993	**7.9994**	7.9992
Splash	R	7.8730	**7.9994**	7.9992	7.9993	7.9993	**7.9994**
	G	7.7675	**7.9994**	**7.9994**	7.9992	7.9993	7.9993
	B	7.7106	**7.9994**	**7.9994**	7.9993	7.9993	7.9993

**Table 11:** Image Correlation.

Image	Channel	Input	Cipher Images				
			PD5H	Ref. [20]	Ref. [24]	Ref. [26]	Ref. [32]
Airplane	R	0.9726	**0.0007**	−0.0010	0.0011	−0.0056	0.0021
		0.9507	0.0007	**−0.0006**	−0.0000	−0.0151	−0.0012
		0.9346	0.0009	**0.0006**	−0.0027	0.0014	−0.0011
	G	0.9425	**−0.0001**	−0.0008	−0.0050	−0.0095	0.0018
		0.9665	−0.0020	0.0021	**0.0015**	0.0133	−0.0018
		0.9312	**0.0012**	0.0014	**−0.0012**	0.0189	0.0026
	B	0.9633	0.0028	−0.0012	**0.0011**	−0.0025	0.0020
		0.9162	0.00021	0.0003	0.0037	−0.0159	**0.0001**
		0.911	0.0010	**−0.0002**	−0.0003	−0.0001	−0.0020
Baboon	R	0.9218	0.0007	**−0.0002**	0.0033	0.0018	0.0025
		0.8624	**0.0008**	0.0010	−0.0013	0.0038	0.0020
		0.8531	0.0003	**−0.0001**	−0.0009	−0.0016	0.0000
	G	0.8643	**0.0003**	−0.0008	0.0018	−0.0013	−0.0018
		0.7591	**0.0002**	−0.0017	0.0004	0.0176	−0.0005
		0.7299	−0.0007	−0.0016	**−0.0003**	0.0040	0.0015
	B	0.9071	0.0013	0.0017	**−0.0005**	−0.0112	−0.0019
		0.8782	**−0.0002**	0.0010	−0.0004	0.0018	0.0024
		0.8411	**−0.0005**	0.0016	**−0.0005**	0.0082	0.0013
Peppers	R	0.9618	0.0010	0.0008	**0.0002**	0.0054	0.0019
		0.9640	−0.0013	0.0013	−0.0011	−0.0042	**0.0005**
		0.9575	**0.0020**	0.0037	−0.0024	−0.0177	−0.0024
	G	0.9777	**−0.0001**	0.0028	−0.0003	−0.0055	0.0027
		0.9771	**−0.0009**	0.0021	0.0044	0.0119	0.0035
		0.9698	0.0012	0.0007	**−0.0003**	0.0046	−0.0008
	B	0.9628	**−0.0012**	−0.0015	0.0034	−0.0021	0.0014
		0.9619	0.0007	**0.0002**	0.0007	0.0104	0.0017
		0.9478	−0.0008	−0.0007	**−0.0003**	−0.0021	**0.0003**
Sailboat	R	0.9544	0.0028	**−0.0001**	−0.0006	−0.0025	0.0021
		0.9529	−0.0009	**−0.0005**	0.0011	−0.0092	−0.0048
		0.9396	−0.0028	**0.0000**	−0.0023	−0.0095	−0.0024
	G	0.9692	−0.0030	−0.0042	**0.0006**	0.0124	0.0023
		0.9627	0.0013	**0.0008**	0.0013	0.0102	−0.0012
		0.9520	0.0041	−0.0011	**−0.0003**	−0.0057	−0.0013
	B	0.9690	0.0005	0.0007	**−0.0001**	−0.0073	0.0032
		0.9688	**−0.0015**	0.0022	0.0019	0.0135	−0.0016
		0.9521	0.0026	−0.0025	**0.0001**	0.0034	−0.0006
Splash	R	0.9936	**0.0009**	0.0026	0.0021	−0.0073	−0.0029
		0.9946	−0.0014	**−0.0001**	−0.0012	0.0136	−0.0028
		0.9893	−0.0011	−0.0011	−0.0007	−0.0006	**0.0004**
	G	0.9796	−0.0010	**0.0000**	0.0025	−0.0066	−0.0025
		0.9831	0.0012	0.0019	**−0.0003**	0.0202	0.0008
		0.9712	−0.0034	−0.0005	**0.0002**	−0.0075	0.0009
	B	0.9626	**0.0008**	0.0038	0.0030	−0.0026	0.0038
		0.9700	**0.0002**	0.0013	−0.0025	0.0023	−0.0037
		0.9653	**0.0001**	−0.0004	−0.0011	−0.0045	0.0013

**Table 12:** The NPCR (%) of the testing images.

Image	Channel	Cipher Images				
		PD5H	Ref. [20]	Ref. [24]	Ref. [26]	Ref. [32]
Airplane	R	99.6216	99.6056	99.6045	99.6063	99.6118
	G	99.6204	99.6116	99.6125	99.6033	99.6107
	B	99.6033	99.6138	99.6233	99.6029	99.6184
	Average	99.6151	99.6103	99.6134	99.6042	99.6136
Baboon	R	99.6349	99.6045	99.6029	99.6048	99.6117
	G	99.6040	99.6108	99.6069	99.6059	99.6109
	B	99.6086	99.6191	99.6117	99.6071	99.6094
	Average	99.6159	99.6115	99.6072	99.6059	99.6107
Peppers	R	99.6202	99.6080	99.6144	99.5975	99.6074
	G	99.6357	99.6069	99.6177	99.6052	99.6064
	B	99.6048	99.6041	99.6190	99.6037	99.6048
	Average	99.6202	99.6063	99.6170	99.6021	99.6062
Sailboat	R	99.6010	99.6112	99.6045	99.6089	99.6144
	G	99.6155	99.6070	99.6167	99.5983	99.6069
	B	99.5781	99.6061	99.5990	99.6174	99.6117
	Average	99.5982	99.6081	99.6067	99.6082	99.6110
Splash	R	99.6166	99.6087	99.6135	99.6037	99.6118
	G	99.6155	99.6112	99.6110	99.6020	99.6114
	B	99.6189	99.6050	99.5993	99.6082	99.6078
	Average	99.6170	99.6083	99.6079	99.6046	99.6103

**Table 13:** The UACI (%) of the testing images.

Image	Channel	Cipher Images				
		PD5H	Ref. [20]	Ref. [24]	Ref. [26]	Ref. [32]
Airplane	R	33.4429	33.4938	33.4393	31.9665	33.4655
	G	33.4552	33.4756	33.4644	33.1449	33.4599
	B	33.4504	33.4568	33.4332	32.7264	33.4647
	Average	33.4495	33.4754	33.4456	32.6126	33.4634
Baboon	R	33.4208	33.4655	33.4285	29.9931	33.4583
	G	33.3322	33.4101	33.4941	28.5822	33.4907
	B	33.4383	33.4822	33.4867	31.2384	33.4578
	Average	33.3971	33.4526	33.4698	29.9379	33.4689
Peppers	R	33.4247	33.4564	33.4579	29.0588	33.4537
	G	33.4939	33.4727	33.4696	33.4382	33.4811
	B	33.5031	33.4897	33.4905	33.4001	33.3860
	Average	33.4739	33.4729	33.4727	31.9657	33.4402
Sailboat	R	33.4384	33.4386	33.4763	27.9264	33.4532
	G	33.4770	33.4555	33.4765	33.4203	33.4721
	B	33.4418	33.4438	33.4591	33.4025	33.4793
	Average	33.4524	33.4460	33.4706	31.5831	33.4682
Splash	R	33.4511	33.5129	33.4592	33.4427	33.4724
	G	33.3700	33.4478	33.4771	33.4605	33.4659
	B	33.4266	33.4273	33.4828	31.9747	33.4741
	Average	33.4159	33.4627	33.4730	32.9593	33.4708

**Table 14:** Encryption and decryption time.

	3 × 128 × 128	3 × 256 × 256	3 × 512 × 512
Encryption Time(s)	0.3143	0.7711	3.0342
Decryption Time(s)	0.3406	0.8188	3.3320

## Data Availability

Data is contained within the article.
